# Aerobic exercise inhibits GSDME-dependent myocardial cell pyroptosis to protect ischemia-reperfusion injury

**DOI:** 10.1186/s10020-024-01048-7

**Published:** 2024-12-24

**Authors:** Yi Li, Xiang Wang, Xuyang Meng, Chenxi Xia, Chenguang Yang, Jun Wang, Jiefu Yang, Fang Wang

**Affiliations:** 1 Department of Cardiology, Beijing Hospital, National Center of Gerontology, Institute of Geriatric Medicine, Chinese Academy of Medical Sciences, Beijing, 100730 People’s Republic of China; 2 Chinese Academy of Medical Sciences and Peking Union Medical College, Beijing, 100730 People’s Republic of China

**Keywords:** Acute myocardial infarction, Ischemia-reperfusion, GSDME, IGFBP2, Pyroptosis, AKT-GSK3β

## Abstract

**Background:**

Acute myocardial infarction (AMI) remains a significant cause of global mortality, exacerbated by ischemia-reperfusion (IR) injury. Myocardial cell pyroptosis has emerged as a critical pathway influencing IR injury severity.

**Methods:**

We aimed to investigate the cardioprotective effects of aerobic exercise on IR injury by examining the modulation of IGFBP2 and its impact on GSDME-dependent myocardial cell pyroptosis. Mechanistic pathways were explored using western blot analysis, ELISA, immunofluorescence, and echocardiography.

**Results:**

Our findings demonstrate that aerobic exercise leads to increased circulating levels of IGFBP2, which effectively suppresses GSDME-dependent myocardial cell pyroptosis. This regulation occurs via the AKT-GSK3β signaling pathway, involving VDAC1 phosphorylation, thereby enhancing mitochondrial function and reducing oxidative stress.

**Conclusion:**

In conclusion, our study highlights the role of IGFBP2 in mitigating GSDME-dependent pyroptosis as a mechanism through which aerobic exercise exerts cardioprotective effects against IR injury. These insights suggest potential therapeutic targets for managing acute myocardial infarction.

## Introduction

Acute myocardial infarction (AMI) is a leading cause of morbidity and mortality worldwide, affecting millions annually (Antithrombotic Trialists’ Collaboration 2002). According to the World Health Organization, cardiovascular diseases (CVDs), including AMI, account for approximately 17.9 million deaths each year, with a significant proportion attributed to ischemia-reperfusion (IR) injury following therapeutic interventions (Vaduganathan et al. 2022). Timely reperfusion therapy remains the cornerstone of AMI treatment; however, IR injury poses a significant challenge by exacerbating myocardial damage and limiting the efficacy of these treatments (Bugger and Pfeil 2020; Xu et al. 2023; An et al. 2022). Despite advancements in therapeutic strategies, effective prevention and treatment of IR injury remain elusive.

Emerging evidence highlights the critical role of programmed cell death (PCD) in myocardial IR injury. Apoptosis, pyroptosis, and ferroptosis have been identified as major contributors to IR injury (Bei et al. 2022). Notably, myocardial pyroptosis, mediated by the NLRP3 inflammasome and Gasdermin D (GSDMD), has been shown to impair cardiac function during IR injury (Davidson et al. 2020; Li et al. 2022a). Inhibiting pyroptosis through targeted pathways has demonstrated significant cardioprotective effects. Similarly, suppressing ferroptosis—a form of iron-dependent cell death—has been reported to restore cardiac function post-IR injury (Cai et al. 2023; Ding et al. 2023; Wang et al. 2023; Yanpiset et al. 2023). Collectively, these findings underscore the importance of understanding the molecular mechanisms underlying myocardial cell death to identify novel therapeutic targets.

Recent studies have identified the AKT-GSK3β signaling pathway as a critical regulator of cell survival and death in the context of CVDs. AKT activation inhibits GSK3β, a kinase implicated in promoting apoptosis and pyroptosis under stress conditions. Additionally, AKT-GSK3β signaling modulates the phosphorylation of voltage-dependent anion channel 1 (VDAC1), a key protein in mitochondrial function and cell death regulation. Dysfunctional VDAC1 has been associated with mitochondrial permeability transition pore (mPTP) opening, exacerbating IR injury (Abeyrathna and Su 2015; Ajzashokouhi et al. 2023; Ghafouri-Fard et al. 2022; Deng and Zhou 2023). Targeting this pathway offers a promising avenue for mitigating IR-induced myocardial damage.

Aerobic exercise has emerged as a non-pharmacological intervention with profound cardioprotective effects. It is known that exercise plays an important protective role in cardiovascular health, as evidenced by several studies (Penna et al. 2020; Gao et al. 2023; Zhang et al. 2024). The reduction of pyroptosis, intended as inflammasome activation, appears to be mediated by exercise, as indicated by a study focusing on one aspect of this relationship (Ding et al. 2024). Long-term aerobic exercise is known to upregulate insulin-like growth factor-binding protein 2 (IGFBP2), which has been linked to improved cardiac outcomes in AMI patients. IGFBP2 not only enhances myocardial resilience but also inhibits pyroptosis by modulating key signaling pathways (Sterczala et al. 2022; Mirna et al. 2020). Preliminary findings from our studies suggest that IGFBP2 may inhibit GSDME-dependent pyroptosis through the AKT-GSK3β signaling pathway by regulating VDAC1 phosphorylation, thus protecting against IR injury.

This research aims to elucidate the role of IGFBP2 in mediating the cardioprotective effects of aerobic exercise in IR hearts. Specifically, we will investigate its potential to inhibit GSDME-dependent pyroptosis via AKT-GSK3β-VDAC1 signaling. By uncovering these mechanisms, our findings may pave the way for innovative therapeutic strategies targeting myocardial IR injury, ultimately improving patient outcomes in CVD.

## Method and materials

### Ethics statement

This study was performed following the approval of the Ethical Committee of Peking Union Medical College Hospital. All animal experiments were implemented based on the Guide for the Care and Use of Laboratory Animals.

### Mice

All C57BL/6 male mice, aged 6–8 weeks and weighing approximately 22 ± 1.5 g, were purchased from Shanghai Slac Laboratory Animal Co., Ltd. (Shanghai, China). The animals were housed under specific pathogen-free (SPF) conditions at a controlled temperature of 23 °C with 65% humidity and maintained on a 12-hour light/dark cycle. Food and water were provided ad libitum. For each experimental group, at least eight mice were used to ensure statistical reliability. Animals were randomly assigned to experimental groups. No animals were excluded from the study unless predefined exclusion criteria, such as illness unrelated to the experimental procedures or unexpected mortality, were met. However, no exclusions were necessary during the course of this study. *Igfbp2*^*−/−*^ and *Myh6*^cre^*Gsdme*^flox/flox^ (GSDMEcKO) crossed to produce first-generation mice with the desired genotypes (*Igfbp2*^+/−^, *Gsdme*^+/−^, Cre^+/−^). Subsequently, these first-generation mice were bred separately with *Igfbp2*^−/−^ mice and GSDMEcKO mice to obtain second-generation mice with the genotypes *Igfbp2*^−/−^, *Gsdme*^+/−^, Cre^+/−^, and *Igfbp2*^+/−^, *Gsdme*^−/−^, Cre^+/+^. Finally, these two groups of second-generation mice were crossed to obtain double knockout mice with the genotype *Igfbp2*^−/−^*Myh6*^cre^*Gsdme*^flox/flox^ (IGFBP2KO GSDMEcKO). This breeding strategy ensures the generation of mice with the desired gene knockout combinations for further experimental studies.

### Ischemia-reperfusion (IR) heart injury mouse model

The indicated mice were first anesthetized with intraperitoneal injections of ketamine and xylazine, ensuring adequate depth of anesthesia. A horizontal incision was then made on the chest to expose the heart, followed by ligating the left anterior descending artery (LAD) at its origin and inserting a PE-10 tube between the ligature and the vessel to induce ischemia. Successful induction of ischemia was confirmed by observing the pale coloration of the distal part of the vessel and myocardium. After 2 h of ischemia, the PE-10 tube was removed, and the ligature was loosened to allow for reperfusion. The myocardium, previously pale, began to regain its red coloration, indicating successful reperfusion. Finally, the chest wall was closed, confirming the successful construction of the ischemia-reperfusion model (Xu et al. 2014). This model allows for the study of various aspects of myocardial ischemia-reperfusion injury in mice.

### Aerobic exercise mouse model

The indicated mice underwent a structured aerobic exercise intervention program, conducted from Monday to Friday each week using a modular treadmill. Each aerobic exercise session lasted for 0.5 h, with exercise intensity set at 40–69% of the mouse’s current maximum oxygen consumption (VO2max). Cardiopulmonary exercise testing was performed every Saturday, while Sundays were designated as rest days. Cardiopulmonary exercise testing involved the use of a respiratory metabolic performance testing system combined with the modular treadmill to determine the mice’s maximum oxygen consumption data, representing their "current VO2max" for the upcoming week. The criteria for determining VO2max included observing a plateau in VO2max where oxygen consumption no longer increased with increasing workload or showed a change of less than 5%. In the absence of a VO2max plateau, the peak oxygen consumption value was considered as VO2max (Brossia-Root et al. 2016).

### Myocardial hypoxia/reoxygenation cell in vitro experiment

C57BL/6 pregnant mice were euthanized, and fetal mouse hearts were collected under SPF conditions. The hearts were minced in ice-cold PBS, washed with 1640 medium, and then cultured in 1640 medium containing 10% FBS, 1% antibiotics, and 50ng/mL MCSF at 5% CO2 and 37 °C to obtain cardiomyocytes. The hypoxia/reoxygenation cell model was created by culturing the cardiomyocytes in DMEM medium under anaerobic conditions for 10 hours, followed by a switch to normal DMEM medium and culturing under 95% air, 5% CO_2_, and 37 °C for 24 h (Cao et al. 2019). This model allows for the study of cellular responses to hypoxia and reoxygenation in vitro.

### Triphenyltetrazolium chloride (TTC) staining

The infarct size was determined using TTC staining (Solarbio Life Science, #G3005). Following sacrifice, hearts were rapidly excised and mounted on a Langendorff apparatus for perfusion with Krebs-Henseleit buffer at 37 °C to clear blood and ensure uniform staining. Hearts were then arrested in diastole by injecting 0.5 mL of 10% KCl solution into the left ventricle and sliced into 2 mm thick transverse sections from apex to base. These sections were incubated in 1% TTC solution in phosphate-buffered saline (PBS) at 37 °C for 15 min in the dark to stain the viable myocardium red. The area at risk (AAR) and the infarct area were identified by visual inspection, with the AAR defined as the pale-colored area and the infarct area as the unstained white region. Infarct size was quantified using image analysis software (e.g., ImageJ) to calculate the area of each slice that was not stained by TTC and expressed as a percentage of the total left ventricular area, with the mean value obtained per heart.

### Western blot

Total protein was extracted from myocardial tissues, and its concentration was determined using the bicinchoninic acid kit (BOSTER Biological Technology Co., Ltd., Wuhan, Hubei, China). The protein underwent separation through 10% polyacrylamide gel electrophoresis and was transferred onto polyvinylidene fluoride membranes. These membranes were then incubated with 5% BSA and specific primary antibodies against GSDMD (ab209845, 1:1000, Abcam), cleaved caspase-1 (YC0002, 1:1000, Immunoway, Suzhou, Jiangsu, China), and β-actin (ab5694, 1:1000, Abcam) overnight at 4 °C. Subsequently, the membranes were probed with an HRP-conjugated goat anti-rabbit IgG secondary antibody (ab205718, 1:2000, Abcam) for 2 hours and visualized using an enhanced chemiluminescence system. Signal density analysis was performed using NIH Image J software, with β-actin serving as an internal reference for normalization.

### Immunofluorescence

Immunofluorescence staining was performed according to the standard protocol. Briefly, the cells were fixed with 4% paraformaldehyde, permeabilized with 0.2% Triton X-100, and blocked with 5% BSA at room temperature for 1 h. Then, cells were incubated with Tunnel staining solution (ab66110, Abcam), endoplasmic reticulum (ER)-Tracker™ Red (E34250, ThermoFisher), Hoechst 33342 (62249, ThermoFisher) and DAPI (D1306, ThermoFisher) for 1 h at 37 °C in the dark. The images were observed under the microscope (Zeiss).

### Statistical analysis

Statistical analysis was conducted using SPSS 21.0 (IBM Corp., Armonk, NY, USA) and GraphPad Prism 8.0 (GraphPad Software Inc., San Diego, CA, USA) for data analysis and plotting. Data are represented as mean ± standard deviation. The t-test was used for comparing two groups, while one-way or two-way analysis of variance (ANOVA) was utilized for comparisons among multiple groups, followed by Tukey’s multiple comparison test. Results were considered significant at a p-value < 0.05.

## Results

### Aerobic exercise inhibits GSDME-dependent myocardial cell pyroptosis by elevating peripheral circulating IGFBP2 levels

**Fig. 1:**
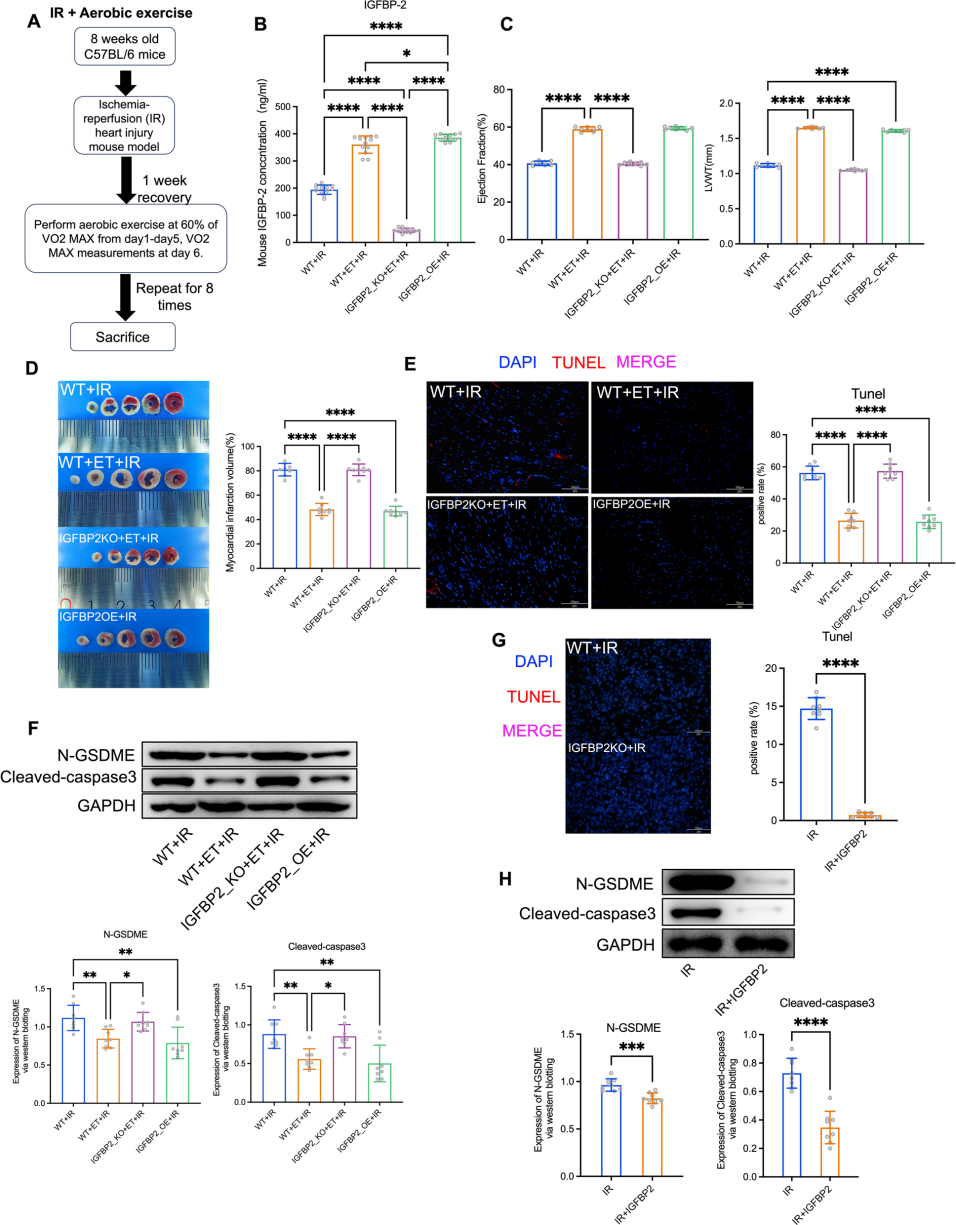
Aerobic exercise inhibits myocardial cell pyroptosis by elevating peripheral circulating IGFBP2 levels. **A** The diagram presents the protocol for the animal model used in the study. **B** IGFBP2 levels in serum from WT+IR, WT+Exercise+IR, IGFBP2_KO+Exercise+IR, IGFBP2_OE+IR mice measured by ELISA. **C** Echocardiographic results of each mouse group. **D** TTC staining results of mouse cardiac tissues from each mouse group. **E** TUNEL staining results of mouse cardiac tissues from each mouse group. **F** Western blot analysis of N-GSDME and cleaved-caspase3 in mouse cardiac tissues. **G** TUNEL staining of cardiomyocytes (IR, IR+IGFBP2). **H** Western blot detection of N-GSDME and cleaved-caspase3 in cardiomyocytes of each group

Initially, we observed elevated IGFBP2 levels in the serum of wild-type (WT) mice but not Igfbp2^−/−^ mice subjected to aerobic exercise prior to ischemia-reperfusion (IR) injury (ET+IR) (Fig. 1A), as measured by ELISA. Additionally, we established IGFBP2 overexpression (OE) in mice, resulting in significantly higher levels of serum IGFBP-2 (Fig. 1B). Echocardiographic assessments demonstrated improved cardiac function in WT but not *Igfbp2*^*−/−*^ mice after aerobic exercise. Furthermore, TTC staining of cardiac tissues confirmed reduced infarct sizes in WT mice after aerobic exercise, as well as in Igfbp2 OE mice without aerobic exercise, but not in Igfbp2^−/−^ mice, underscoring the protective effects of IGFBP2 against myocardial damage (Fig. 1C and D). Mechanistically, TUNEL staining and Western blot analysis revealed reduced myocardial cell death and pyroptosis markers (cleaved-caspase3) in WT mice after aerobic exercise and *Igfbp2* OE group compared to control groups (Fig. 1E–H). Additionally, we found that aerobic exercise and *Igfbp2* OE could reduce the levels of N-terminal fragment of gasdermin E (N-GSDME) in myocardial cells, suggesting the involvement of GSDME in IGFBP2-mediated regulation of myocardial cell pyroptosis (Fig. 1F and H).

**Fig. 2:**
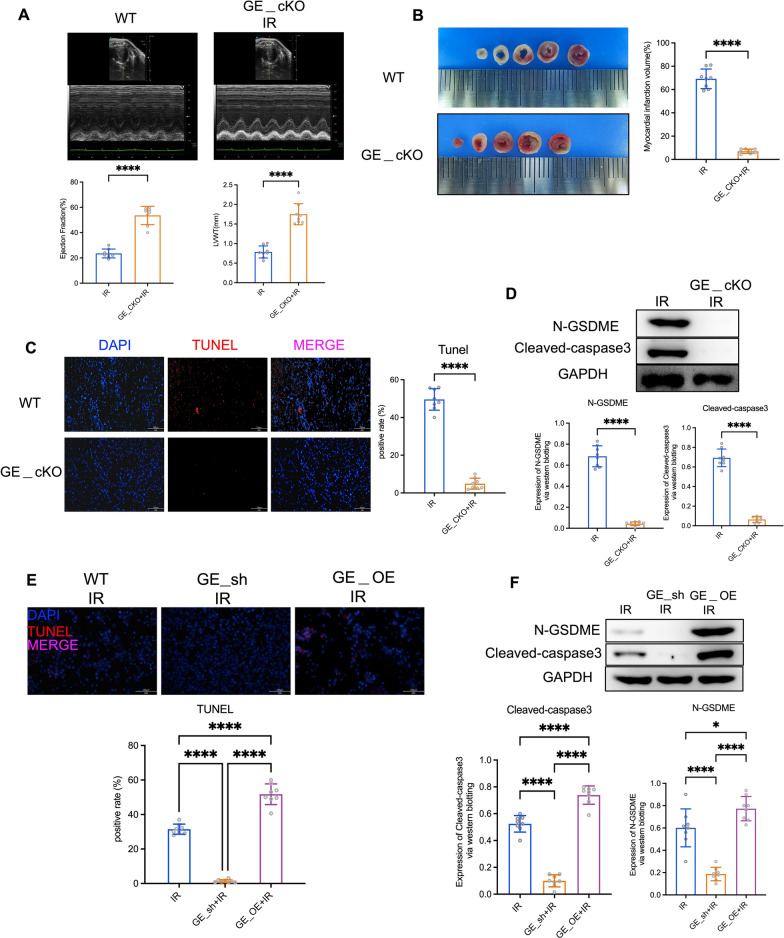
The GSDME-dependent myocardial cell pyroptosis mediates heart IR injury. **A** Echocardiographic results and left ventricular thickness from control mice and GSDMEcKO (GE___cKO) mice. **B** Triphenyl Tetrazolium Chloride (TTC) staining of heart tissues from control mice and GE___cKO mice. **C** TUNEL staining results of heart tissues from control mice and GE___cKO mice. **D** WB results of N-GSDME and cleaved-caspase3 in heart tissues of control mice and GE___cKO mice. **E** TUNEL staining of WT, GE___sh and GE___OE cardiomyocytes. **F** Western blot analysis results of N-GSDME and cleaved-caspase3 in WT, GE___sh and GE___OE cardiomyocytes

To assess the importance of GSDME in myocardial cell pyroptosis during heart IR injury, we examined heart tissue from WT and GSDME conditional knockout (GSDMEcKO) IR injury models via echocardiography. Our findings revealed improved left ventricular function and reduced infarct size in the GSDMEcKO mice compared to controls (Fig. 2A). Triphenyl Tetrazolium Chloride (TTC) staining corroborated the reduced infarct size in the GSDMEcKO IR model (Fig. 2B). Additionally, TUNEL staining unveiled a decrease in apoptotic cells within the hearts of GSDMEcKO IR model mice (Fig. 2C), aligning with the observed downregulation of cleaved-caspase3 expression as demonstrated by western blot analysis of heart tissues (Fig. 2D). In in vitro experiments, we initiated manipulation of GSDME expression levels to elucidate its role in cardiomyocyte function under hypoxia/reoxygenation conditions. GSDME knockdown (GE_sh) was achieved by introducing shRNA sequences targeting GSDME mRNA into cardiomyocytes. Conversely, GSDME overexpression (GE_OE) was facilitated by the introduction of adenoviruses engineered to carry GSDME cDNA sequences into cardiomyocytes, resulting in GSDME overexpression. Consistent with in vivo observations, TUNEL staining and western blot analysis of WT, GE_sh, and GE_OE cardiomyocytes demonstrated that genetic modulation of GSDME expression regulates cell pyroptosis (Fig. 2E, F). Collectively, these data underscore the necessity of GSDME in cardiomyocyte pyroptosis inhibited by IGFBP-2 during myocardial IR injury.

**Fig. 3:**
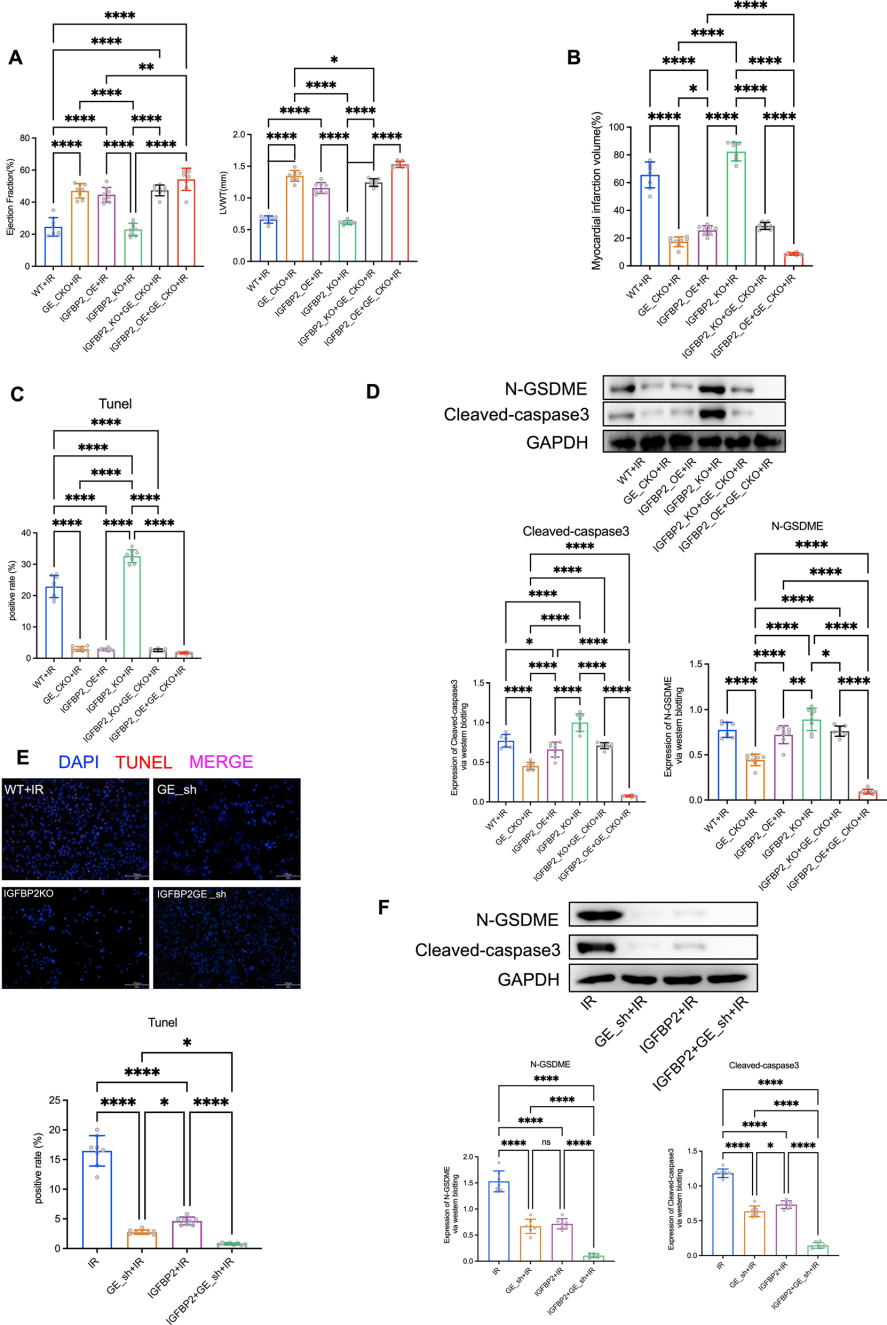
GSDME is required for the apoptosis of myocardial cells inhibited by IGFBP2 in IR heart. **A** Echocardiographic assessment results of mouse experimental groups including WT+ IR, GE_CKO+IR, IGFBP2_OE+IR, IGFBP2_KO+IR, IGFBP2_KO+GE_CKO+IR, IGFBP2_OE+GE_CKO+IR. **B** TTC staining results of mouse cardiac tissues of each mouse group. **C** TUNEL staining results of mouse cardiac tissues. **D** Western blot analysis of N-GSDME and cleaved-caspase3 in mouse cardiac tissues. **E** TUNEL staining of cardiomyocytes in cell experimental groups. **F** Western blot detection of N-GSDME and cleaved-caspase3 in cardiomyocytes of cell experimental groups

Furthermore, echocardiographic, TTC, and TUNEL staining results indicated that IGFBP2 knockout failed to induce myocardial injury and cell death in GSDME-deficient conditions (Fig. 3A–C). Western blot analysis further confirmed the protective effects of IGFBP2 on myocardial cell pyroptosis in a GSDME-dependent manner (Fig. 3D). Additionally, cellular experiments reinforced the cardioprotective effects of IGFBP2 via the reduced GSDME-dependent pyroptosis in cardiomyocytes (Fig. 3E, F).

### IGFBP2 protects mitochondrial function of myocardial cell by upregulating AKT-GSK3β phosphorylation levels

**Fig. 4:**
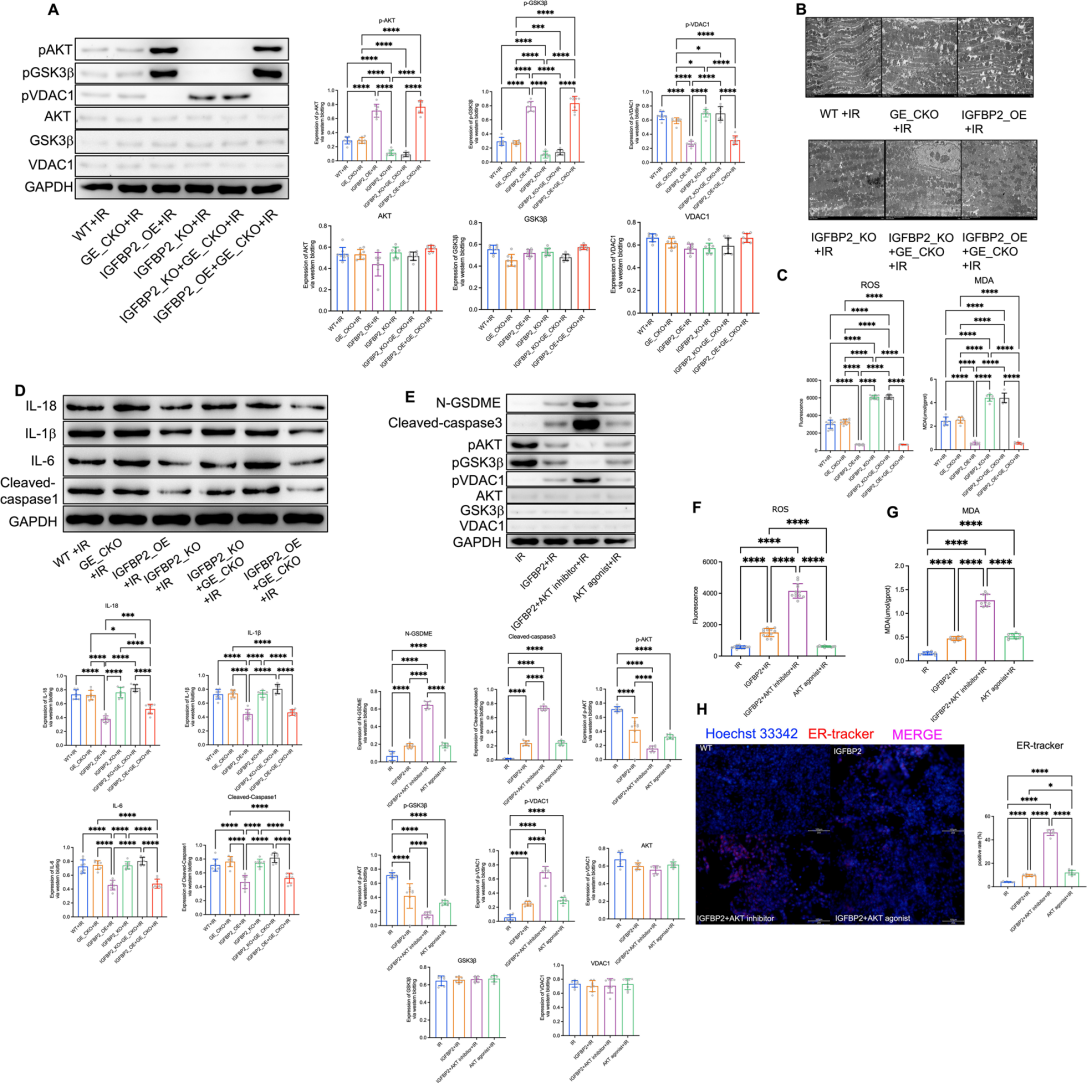
IGFBP2 protects mitochondrial function of myocardial cell by upregulating AKT-GSK3β phosphorylation levels. **A** Western blot analysis of p-AKT, p-GSK3β, p-VDAC1, AKT, GSK3β, and VDAC1 levels in heart tissue from each mouse experimental group. **B** Electron microscopy of mouse heart tissue across experimental groups. **C** Quantification of ROS and MDA levels in mouse heart tissue across experimental groups. **D** Assessment of inflammatory cytokines and cleaved-caspase 1 levels in mouse heart tissue across experimental groups. **E** Western blot analysis of cardiomyocytes from various experimental groups showing protein expression levels of N-GSDME, cleaved-caspase3, p-AKT, p-GSK3β, p-VDAC1, AKT, GSK3β, and VDAC1. **F** Assessment of ROS levels in cardiomyocytes across experimental groups. **G** Measurement of MDA levels in cardiomyocytes across experimental groups. **H** Immunofluorescence staining of cardiomyocytes using ER-tracker (red) and Hoechst 33342 (blue) to visualize mitochondrial respiratory function

Activation of Caspase3 has been shown to trigger GSDME-dependent pyroptosis, and is highly associated with the release of various danger signals within mitochondria (Hu et al. 2023; Li et al. 2022b). Previous studies have suggested that the activated AKT-GSK3β signaling pathway can protect IR cardiomyocytes' mitochondria by dephosphorylating VDAC1, reducing the release of mitochondrial danger signals (Tian et al. 2019), but its effect on GSDME-dependent pyroptosis remains unreported. Therefore, we hypothesized that AKT-GSK3β signaling pathway may be involved in regulatory effect on GSDME-dependent cell pyroptosis in IR hearts. Western blot analysis showed increased levels of phosphorylated AKT, GSK3β, and VDAC1 in IGFBP2 OE mice and decreased levels in *Igfbp2*^−/−^ mice. Moreover, GSDME deficiency didn’t change levels of phosphorylated AKT, GSK3β, and VDAC1 (Fig. 4A). Electron microscopy results suggested the structure of heart tissue of *Igfbp2*^−/−^ is disrupted and their distribution is irregular (Fig. 4B). Additionally, the decrease in reactive oxygen species (ROS) and malondialdehyde (MDA) levels in *Igfbp2* OE mice and the increase in *Igfbp2*^−/−^ mice suggest that IGFBP2 improves oxidative stress management and mitochondrial function in myocardial cells (Fig. 4C). Furthermore, overexpression of IGFBP2 effectively suppresses inflammatory cytokines, such as IL-18, IL-1β and IL-6, and cleaved-caspase 1 to prevent inflammation in IR conditions (Fig. 4D). Further cellular analysis including western blot analysis of phosphorylated AKT, GSK3β, and VDAC1 levels, and immunofluorescence staining of ER-tracker, highlighting IGFBP2's regulation of these pathways at the cellular level, included cellular N-GSDME and cleaved-caspase3 levels and endoplasmic reticulum morphology (Fig. 4E–H).

**Fig. 5:**
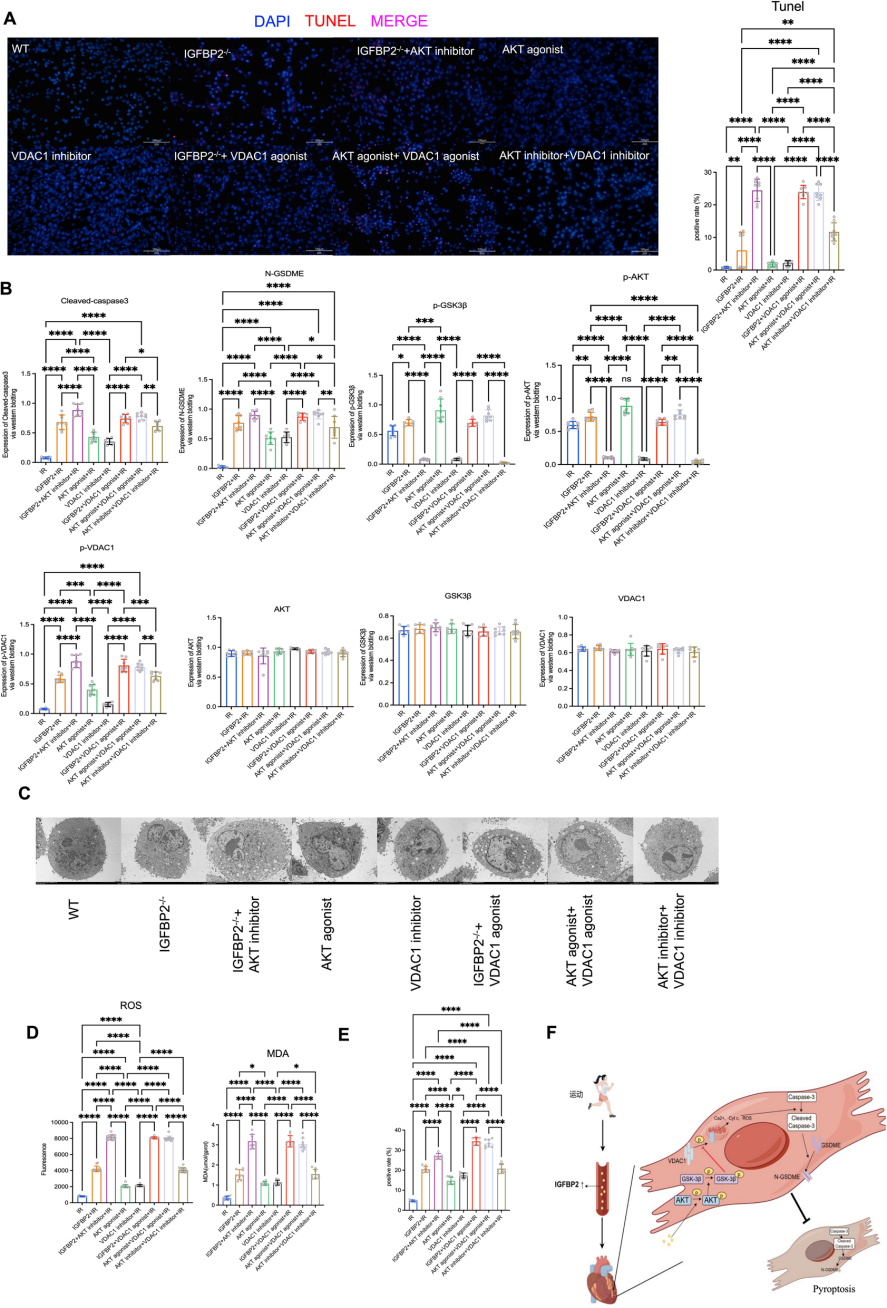
Phosphorylation of VDAC1 inhibits glucose GSDME-dependent pyroptosis by modulating mitochondrial function. **A** TUNEL staining of cardiomyocytes in each experimental group. **B** Western blot analysis of cardiomyocytes from each experimental group showing protein expression levels of N-GSDME, cleaved-caspase3, p-AKT, p-GSK3β, p-VDAC1, AKT, GSK3β, and VDAC1. **C** Electron microscopy of cardiomyocytes from each experimental group. **D** Quantification of ROS and MDA levels in cardiomyocytes across experimental groups. **E** Immunofluorescence staining of cardiomyocytes using ER-tracker (red) and Hoechst 33342 (blue) to visualize mitochondrial respiratory function. **F** A proposed model for this study

Next, we utilized AKT and VDAC1 agonist and inhibitor to determine the roles of AKT and VDAC1 phosphorylation in this regulation. TUNEL staining results showed that AKT inhibitor and VDAC1 agonist increased myocardial cell pyroptosis levels. Moreover, the VDAC1 agonist counteracted the effects of the AKT inhibitor on pyroptosis regulation, suggesting that VDAC1 is downstream of AKT phosphorylation in this pathway. Furthermore, TUNEL and western blot analyses demonstrated reduced pyroptosis in cardiomyocytes with altered AKT and VDAC1 phosphorylation states (Fig. 5A, B). Electron microscopy results revealed that AKT activation protects mitochondria, whereas VDAC1 activation leads to mitochondrial damage (Fig. 5C). Measurements of ROS and MDA levels confirmed the role of mitochondrial function with different treatments, AKT inhibitor and VDAC1 agonist both increase ROS and MDA levels in myocardial cell (Fig. 5D). Immunofluorescence staining provided visual confirmation of improved endoplasmic reticulum structure (Fig. 5E). In summary, our data suggest that aerobic exercise training upregulates circulating IGFBP2 levels, which, by enhancing AKT-GSK3β phosphorylation levels within IR myocardial cells, subsequently downregulates VDAC1 phosphorylation levels, ultimately inhibiting GSDME-dependent apoptosis in myocardial cells and protecting heart function (Fig. 5F).

## Discussion

Cardiac IR injury remains a major clinical challenge, contributing to significant morbidity and mortality worldwide. While substantial progress has been made in understanding the basic mechanisms underlying IR injury, the role of cell death pathways, particularly GSDME-dependent pyroptosis, has been underexplored. Our study fills this critical gap by highlighting the involvement of GSDME in cardiac IR injury and establishing IGFBP2 as a promising cardioprotective mediator.

Our findings identify GSDME-dependent pyroptosis as a central driver of myocardial damage in IR injury. Pyroptosis, a form of inflammatory programmed cell death, exacerbates tissue damage by releasing pro-inflammatory cytokines and damaging cellular membranes. Although previous studies have implicated pyroptosis in various cardiac pathologies, our study is among the first to focus on the specific role of GSDME in myocardial injury. The demonstration of GSDME’s involvement in exacerbating cardiac dysfunction following IR injury provides a mechanistic basis for targeting this pathway in therapeutic strategies. The contribution of GSDME-dependent pyroptosis to mitochondrial dysfunction is particularly notable. Our data suggest that pyroptotic processes not only disrupt cellular integrity but also compromise mitochondrial health, thereby aggravating the energy deficit in stressed myocardial cells. This mechanistic insight into how pyroptosis exacerbates IR injury provides a deeper understanding of its pathogenic role and highlights the urgent need to develop strategies to mitigate this process.

One of the major findings of our study is the identification of IGFBP2 as a cardioprotective factor that mitigates GSDME-dependent pyroptosis. Aerobic exercise was found to elevate peripheral levels of IGFBP2, suggesting a mechanistic link between lifestyle interventions and myocardial protection. Previous research has highlighted the beneficial effects of IGFBP2 in various physiological contexts, but its role in cardiac IR injury has remained unclear. Our study provides robust evidence that IGFBP2 reduces myocardial damage by preserving mitochondrial integrity and modulating pyroptosis pathways. At the molecular level, we demonstrate that IGFBP2 exerts its effects through the AKT-GSK3β signaling axis. Activation of this pathway has been associated with enhanced cell survival and reduced oxidative stress in various tissues. By linking IGFBP2 to this signaling cascade, our findings provide a mechanistic explanation for its cardioprotective effects. Moreover, we establish that IGFBP2 modulates the phosphorylation status of VDAC1, a key mitochondrial protein involved in regulating cellular energy metabolism and apoptosis. This novel insight into the IGFBP2-VDAC1 axis adds an additional layer of complexity to the molecular mechanisms underlying myocardial protection.

Our findings underscore the potential of aerobic exercise as a non-pharmacological intervention to mitigate IR injury. The elevation of circulating IGFBP2 levels following exercise not only highlights the systemic benefits of this intervention but also positions IGFBP2 as a potential biomarker for exercise-induced cardioprotection. These results reinforce the importance of lifestyle modifications in managing cardiac diseases and suggest that exercise could be an adjunct to pharmacological therapies targeting IR injury. The ability of exercise to modulate critical pathways implicated in pyroptosis and mitochondrial dysfunction highlights its potential as a low-cost, widely accessible strategy for reducing the burden of IR injury. Future research should focus on optimizing exercise regimens to maximize IGFBP2 levels and further elucidate the molecular pathways through which exercise exerts its cardioprotective effects.

The identification of IGFBP2 as a key modulator of GSDME-dependent pyroptosis opens new avenues for therapeutic intervention. Targeting IGFBP2 or its downstream pathways could provide a novel approach for protecting the myocardium from IR injury. Moreover, the modulation of VDAC1 phosphorylation represents a potential strategy for preserving mitochondrial function in stressed cardiac cells. Our findings also raise the possibility of developing pharmacological agents that mimic the effects of IGFBP2 or enhance its expression. Such agents could complement existing therapies for IR injury and improve clinical outcomes. Additionally, the involvement of the AKT-GSK3β pathway in mediating the effects of IGFBP2 suggests that drugs targeting this pathway could have synergistic effects with IGFBP2-based therapies.

Beyond cardiac IR injury, the insights gained from this study have broader implications for other conditions characterized by pyroptosis and mitochondrial dysfunction. Diseases such as stroke, neurodegenerative disorders, and metabolic syndromes may also benefit from interventions targeting the pathways identified in our study. Furthermore, the role of IGFBP2 in regulating cellular energy metabolism and inflammation warrants further investigation in these contexts. Future studies should also explore the translational potential of our findings. Clinical trials investigating the efficacy of IGFBP2-based therapies or exercise regimens in patients with cardiac IR injury would be a logical next step. Additionally, the development of biomarkers for GSDME-dependent pyroptosis could facilitate the early detection and monitoring of IR injury.

## Conclusion

In conclusion, our study provides compelling evidence for the role of GSDME-dependent pyroptosis in cardiac IR injury and highlights IGFBP2 as a key mediator of myocardial protection. By elucidating the molecular pathways linking IGFBP2 to mitochondrial function and pyroptosis regulation, we have identified novel therapeutic targets that could transform the management of cardiac IR injury.

The cardioprotective effects of aerobic exercise, mediated in part by IGFBP2, underscore the potential of lifestyle interventions in mitigating myocardial damage. These findings not only advance our understanding of the pathophysiology of IR injury but also pave the way for the development of innovative therapies. Whether through pharmacological agents, exercise regimens, or a combination of both, targeting the IGFBP2-AKT-GSK3β-VDAC1 axis holds promise for improving outcomes in patients with cardiac IR injury. This work emphasizes the critical interplay between cellular signaling, mitochondrial health, and systemic interventions, providing a foundation for future research and therapeutic innovation.

## Data Availability

No datasets were generated or analysed during the current study.
